# Omega-3 Polyunsaturated Fatty Acids Attenuate Fibroblast Activation and Kidney Fibrosis Involving MTORC2 Signaling Suppression

**DOI:** 10.1038/srep46146

**Published:** 2017-04-10

**Authors:** Zhifeng Zeng, Haiyuan Yang, Ying Wang, Jiafa Ren, Yifan Dai, Chunsun Dai

**Affiliations:** 1 Center for Kidney Diseases, 2nd Affiliated Hospital, Nanjing Medical University, 262 North Zhongshan Road, Nanjing, Jiangsu, China; 2 Jiangsu Key Laboratory of Xenotransplantation, Nanjing Medical University, Nanjing, Jiangsu 210029, China

## Abstract

Epidemiologic studies showed the correlation between the deficiency of omega-3 polyunsaturated fatty acids (n-3 PUFAs) and the progression of chronic kidney diseases (CKD), however, the role and mechanisms for n-3 PUFAs in protecting against kidney fibrosis remain obscure. In this study, NRK-49F cells, a rat kidney interstitial fibroblast cell line, were stimulated with TGFβ1. A Caenorhabditis elegans fat-1 transgenic mouse model in which n-3 PUFAs are endogenously produced from n-6 PUFAs owing to the expression of n-3 fatty acid desaturase were deployed. Docosahexaenoic acid (DHA), one member of n-3 PUFAs family, could suppress TGFβ1-induced fibroblast activation at a dose and time dependent manner. Additionally, DHA could largely inhibit TGFβ1-stimulated Akt but not S6 or Smad3 phosphorylation at a time dependent manner. To decipher the role for n-3 PUFAs in protecting against kidney fibrosis, fat-1 transgenic mice were operated with unilateral ureter obstruction (UUO). Compared to the wild types, fat-1 transgenics developed much less kidney fibrosis and inflammatory cell accumulation accompanied by less p-Akt (Ser473), p-Akt (Thr308), p-S6 and p-Smad3 in kidney tissues at day 7 after UUO. Thus, n-3 PUFAs can attenuate fibroblast activation and kidney fibrosis, which may be associated with the inhibition of mTORC2 signaling.

Chronic kidney diseases (CKD) with increasing prevalence as well as extremely high modality to end stage renal disease have been recognized as a global health problem. Kidney fibrosis, one of the major pathologic characteristics of CKD, is the common pathway leading to end stage renal disease^1^. Although numerous studies pursuing efficient pharmacologic approaches for retarding kidney fibrosis have been made during the past decades, very few reagents are available for patients with CKD currently.

Polyunsaturated acids (PUFAs) are the principal composition of lipid which forms cellular membrane as well as second signaling messengers in mammal cells^2^. PUFAs are synthesized from the saturated fatty acid by desaturase and elongation enzyme. In mammals, PUFAs, especially n-3 PUFAs must be taken from diets due to lacking desaturase. Among PUFAs, n-3 and n-6 are two predominant, structurally and functionally distinctive PUFAs^3^,^4^,^5^. It was reported that human beings selected and evolved on an ideal diet with the n-3/n-6 PUFAs ratio of 1:1 before the agricultural revolution^6^. Unfortunately, this ratio has been changed to 1:20 over the past 150 years due to the less n-3 and excess n-6 PUFAs in our modern diets^7^,^8^. Regarding the critical role for n-3 PUFAs in regulating organ development and function, the deficiency of n-3 PUFAs and the low n-3/n-6 ratio are detrimental for the healthy in human beings^9^,^10^,^11^,^12^,^13^.

Fatty acids are the principal fuel source for the kidneys and the disturbance of fatty acid metabolism may lead to kidney damage^14^. The renal protective effect for n-3 PUFAs is inconsistency among the diet manipulation studies. Many epidemiologic and animal studies show the correlation of n-3 PUFAs deficiency and CKD progression^15^,^16^,^17^,^18^,^19^,^20^. And PUFAs may interfere with renal fibrogenesis via inhibiting pro-inflammatory cytokine production as well as renin and nitric oxide (NO) systems^21^. In polycystic kidney disease (PKD), dietary PUFAs have no effect on retarding disease progression^22^. In kidney tubular cells, Oleate plus human albumin stimulates fibronectin production, which may promote kidney fibrosis^23^,^24^. Large variation for the amount and sources of n-3 PUFAs as well as the rapid oxidation of PUFAs in diets may account for the discrepancy of PUFAs in treating patients or animals with CKD. Fortunately, a mouse model with fatty acid metabolism-1 (fat-1) transgene encoding n-3 fatty acid desaturase was created^25^. In the transgenics, the abundance of n-3 PUFAs is robustly increased accompanied by a decrease of n-6 PUFAs and as a consequence, the ratio of n-6/n-3 is largely decreased compared to wild type mice^25^.

In this study, we found that Docosahexaenoic acid (DHA), one member of n-3 PUFAs, could largely abolish TGFβ1-induced fibroblast activation accompanied by a reduction of Akt but not S6 or Smad3 phosphorylation in NRK-49F cells. Additionally, fat-1 transgenics were resistant to kidney fibrosis and inflammation after unilateral ureter obstruction (UUO). This study suggests that n-3 PUFAs protect against kidney fibroblast activation and kidney fibrosis, which may be associated with the inhibition of mTORC2 signaling pathway.

## Results

### DHA diminishes TGFβ1-induced kidney fibroblast activation

**Figure 1:**
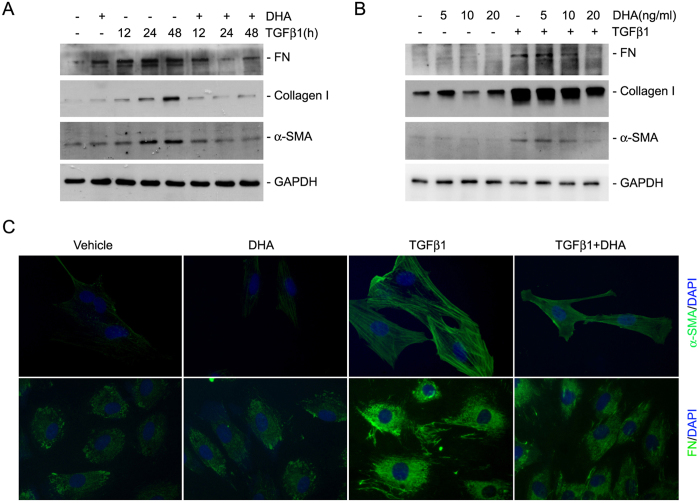
DHA inhibits fibroblast activation induced by TGFβ1. (**A**) Western blotting analysis showing that DHA could time-dependently inhibit the expression of FN, α-SMA and type I collagen stimulated by TGFβ1 treatment. (**B**) Western blotting analysis showing that DHA could dose-dependently inhibit TGFβ1-induced FN, type I collagen and α-SMA expression. (**C**) Representative micrographs showing the immune-staining for FN and α-SMA in NRK-49F cells.

Fibroblast activation plays an essential role for extracellular matrix deposition in the fibrotic kidneys. Here, we treated NRK-49F cells with DHA, a member of n-3 PUFAs family, for 30 minutes, followed by TGFβ1 treatment for 12, 24 and 48 hours. We found that TGFβ1 (2 ng/ml) could stimulate NRK-49F cell activation including the up regulation of α-SMA, fibronection and type I collagen expression. DHA dramatically abrogated TGFβ1-induced fibroblast activation at a time and dose-dependent manner (Fig. 1A,B). Immunofluorescent staining further confirmed the results of western blotting assay (Fig. 1C). These results demonstrate that DHA can efficiently inhibit TGFβ1-induced kidney fibroblast activation.

### DHA inhibits TGFβ1-stimulated Akt phosphorylation in NRK-49F cells

**Figure 2:**
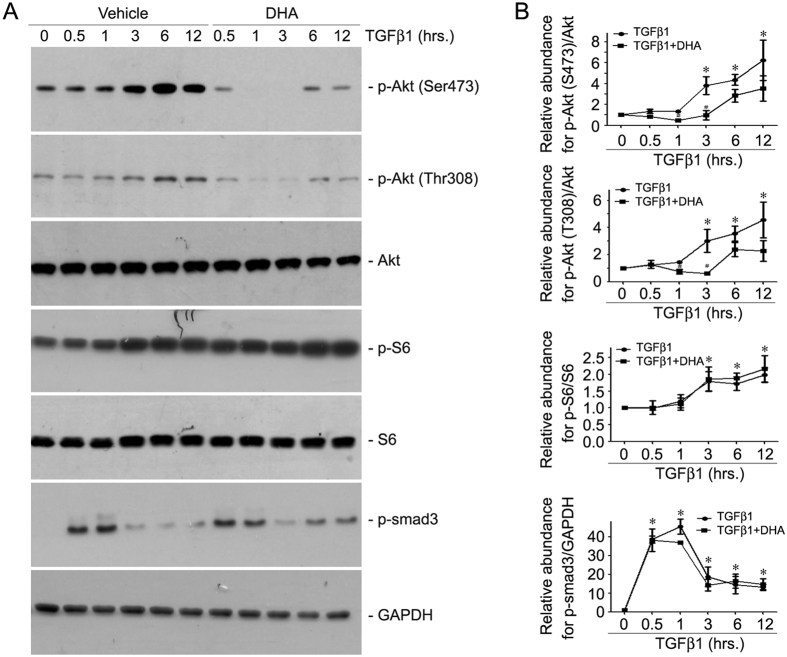
DHA inhibits TGFβ1-stimulated Akt phosphorylation in NRK-49F cells. Cultured NRK-49F cells were serum-starved for 24 h, then administrated with 20 uM DHA for 30 min, followed by TGFβ1 (2 ng/ml) treatment for various time points as indicated. (**A**) Western blotting analyses showing that DHA treatment could decrease the abundance of p-Akt (Ser473) and p-Akt (Thr308) but not p-S6 or p-Smad3 induced by TGFβ1 in NRK-49F cells. (**B**) Quantitative analysis for p-Akt (Ser473), p-Akt (Thr308), p-S6 and p-Smad3. *p < 0.05 vs. control, n = 3; ^#^*P* < 0.05 vs. cells treated with TGFβ1 at the same time point, n = 3.

To further explore the mechanisms for n-3 PUFAs in inhibiting TGFβ1-induced fibroblast activation, we treated NRK-49F cells with DHA w/o TGFβ1 for different time points as indicated and examined the major pro-fibrotic signaling pathways. TGFβ1 could time-dependently stimulate the activation of profibrotic signaling pathways including p-Akt (Ser473), p-Akt (Thr308), p-S6 and p-Smad3 in NRK-49F cells. DHA could markedly inhibit Akt phosphorylation at a time-dependent manner, while p-S6 and p-Smad3 remained unchanged (Fig. 2A,B). These results suggest that DHA may inhibit fibroblast activation via suppressing mTORC2 signaling pathway.

### Fat-1 transgene ameliorates kidney fibrosis in mice with UUO nephropathy

**Figure 3:**
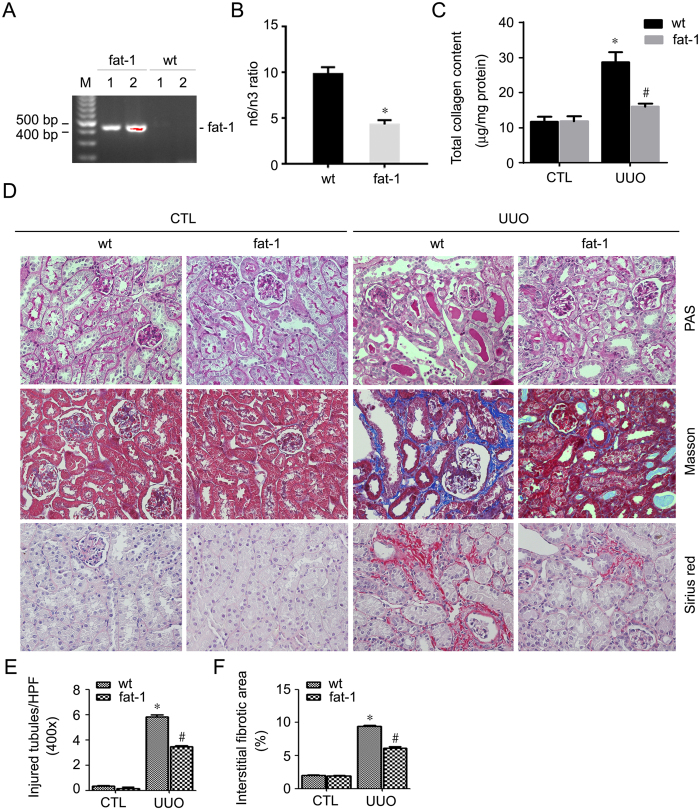
Fat-1 transgenics are resistant to UUO nephropathy. The wild-type and fat-1 transgenic mice received UUO operation and were killed at 7d after surgery. (**A**) PCR image for genotyping. Number 1 and 2 indicate fat-1 transgenics, number 3 and 4 indicate wild type mice. (**B**) Graph showing the ratio of n-6/3 in wild type and fat-1 transgenics. *P < 0.01 vs. wild types, n = 5. (**C**) Graph showing total collagen content in the kidneys from wild type and fat-1 transgenics. *p < 0.05 vs. contra-lateral kidneys, n = 6; ^#^*P* < 0.05 vs. wild type UUO kidneys, n = 4–6. (**D**) Representative micrographs for PAS, Masson and Sirius red staining. (**E**) Graph showing tubule injury in the kidneys from wild type and fat-1 transgenics. *p < 0.05 vs. contra-lateral kidneys, n = 6; ^#^*P* < 0.05 vs. wild type UUO kidneys, n = 4–6. (**F**) Graph showing fibrotic area in the kidneys from wild type and fat-1 transgenics. *p < 0.05 vs. contra-lateral kidneys, n = 6; ^#^*P* < 0.05 vs. wild type UUO kidneys, n = 4–6.

Previous studies demonstrated that introducing exogenous fat-1 gene in mice results in increased production of n-3 PUFAs as well as decreased n-6 PUFAs abundance compared to the wild types^25^. In order to evaluate the protective effect for n-3 PUFAs in kidney fibrosis, fat-1 transgenics were deployed and subjected to unilateral ureter obstruction (UUO). Similar to the previous reports, the ratio of n-6/n-3 PAFAs was largely decreased in the transgenics compared to those in the wild types (Fig. 3A,B). Total collagen content in the contra-lateral kidneys was comparable between two groups, whereas it was significantly decreased in the UUO kidneys from the transgenics compared to those from the wild types (Fig. 3C). Kidney sections were stained with PAS, Masson and Sirius red. As shown in Fig. 3D. Contra-lateral kidney histology was comparable between the transgenics and wild types. Wild type mice displayed remarkable tubular atrophy at 7d after UUO, whereas much less tubular injury was detected in the fat-1 transgenics compared to wild types after UUO (Fig. 3D,E). Masson and Sirius red staining revealed that extracellular matrix deposition was markedly decreased in the UUO kidneys from fat-1 transgenics compared to those from wild types (Fig. 3D,F).

**Figure 4:**
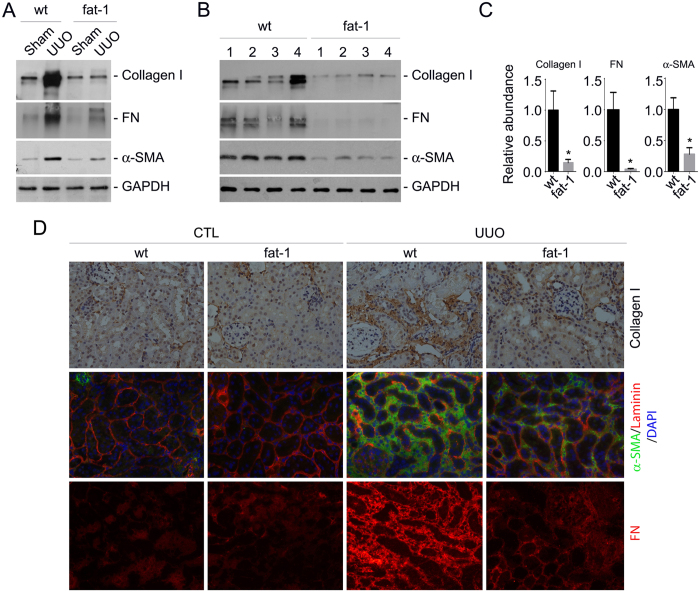
Extracellular matrix production is diminished in the UUO kidneys from *fat-1* transgenics compared to those from wild type mice. (**A**) Western blotting analysis showing FN, α-SMA, and type I collagen within groups as indicated. Kidney samples within each group were pooled together and run the gel. (**B**) Western blotting analysis showing FN, α-SMA, and type I collagen in the fibrotic kidneys from wild type and fat-1 transgenics. Numbers indicate individual animal within each group. (**C**) Graphs showing FN, α-SMA, and type I collagen protein abundance from (**B**). *P < 0.05 vs. wild type UUO kidneys, n = 5–7. (**D**) Representative micrographs showing immune-staining for FN, α-SMA and type I collagen from various groups as indicated.

To further explore the role for fat-1 transgene in protecting against kidney fibrosis, we detected type I collagen, fibronectin and α-SMA expression in the kidneys. Western blotting analysis showed that the induction of collagen I, fibronectin and α-SMA expression in the wild type kidneys after UUO were markedly decreased in the transgenic UUO kidneys (Fig. 4A–C). Immune-staining for type I collagen, fibronectin and α-SMA confirmed the results of western blotting assay (Fig. 4D). Together, these results suggest that fat-1 trangene may protect against UUO nephropathy in mice.

### Fat-1 transgene reduces inflammatory cell accumulation in the kidneys with UUO nephropathy

**Figure 5:**
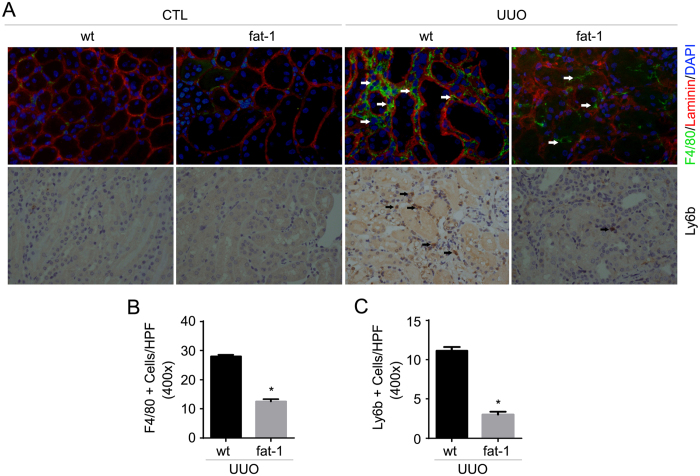
Inflammatory cell accumulation is attenuated in the UUO kidneys from fat-1 transgenics compared to those from wild types. (**A**) Representative micrographs showing immune-staining for F4/80 and Ly6b among different groups as indicated. For F4/80 staining, slides were co-stained with DAPI and Ab against laminin to visualize the cell nuclei and tubular base membrane, respectively. White arrows indicate F4/80 staining positive cells. Black arrows indicate Ly6b staining positive cells. (**B**) The graph showing F4/80-positive cells within the fibrotic kidneys. *P < 0.05 vs. wild type UUO kidneys, n = 4. (**C**) The graph showing Ly6b-positive cells within the fibrotic kidneys. *P < 0.05 vs. wild type UUO kidneys, n = 4.

Inflammatory cell accumulation participates into the initiation and progression of kidney fibrosis. In this study, we examined macrophage and neutrophil accumulation in kidney tissue by immune-staining of F4/80 and Ly6b, respectively. A few F4/80 positive cells were detected in the contra-lateral kidneys from both wild type and fat-1 transgenics. In wild types, F4/80 positive as well as Ly6b positive cells were markedly accumulated in the UUO kidneys compared to the contra-lateral kidneys, while inflammatory cell accumulation was much less in the UUO kidneys from fat-1 transgenics compared to those from the wild types (Fig. 5A–C).

### Fat-1 transgene attenuates the profibrotic signaling activation in the kidneys with UUO nephropathy

**Figure 6:**
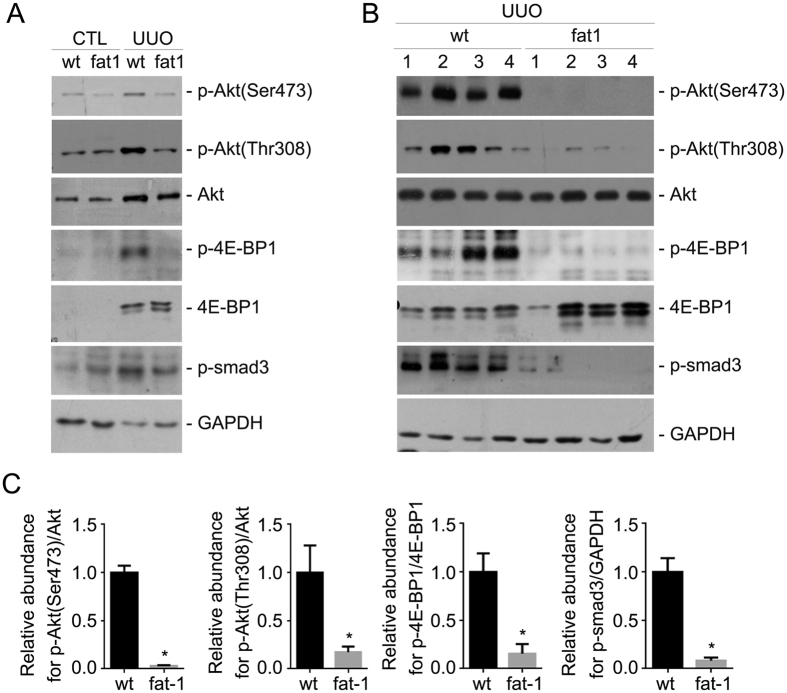
Fat-1 transgene attenuates profibrotic signaling transduction in the UUO kidneys compared to those from the wild types. (**A**) Western blotting analysis showing the abundance for p-Akt (Ser473), p-Akt (Thr308), Akt, p-4E-BP1, 4E-BP1, p-Smad3 and GAPDH in the kidneys from groups as indicated. Kidney samples within each group were pooled together and run the gel. (**B**) Western blot analysis showing the reduction of p-Akt (Ser473), p-Akt (Thr308), p-4E-BP1 and p-Smad3 the UUO kidneys from wild types and fat-1 transgenics. (**C**) Graphs showing p-Akt (Ser473), p-Akt (Thr308), p-4E-BP1 and p-Smad3 abundance within the fibrotic kidneys. *P < 0.05 vs. wild type UUO kidneys, n = 5–7.

To further explore the signaling pathways which may be related to the protective effect for fat-1 transgene in kidneys with UUO nephropathy, we examined mTOR and Smad signaling pathways in kidney tissue from various groups. Western blot analysis shows that Akt, 4E-BP1 and Smad3 phosphorylation were all largely induced in the UUO kidneys compared to the contra-lateral kidneys from the wild type mice (Fig. 6A), whereas the phosphorylation of these three molecules was significantly decreased in the UUO kidneys from fat-1 transgenics compared to those from wild types (Fig. 6B,C).

## Discussion

This study demonstrated that DHA may inhibit TGFβ1-stimulated fibroblast activation. Fat-1 transgene can efficiently protect against kidney fibrosis in mice with UUO nephropathy. Additionally, DHA inhibits Akt but not S6 or Smad3 phosphorylation in NRK-49F cells treated with TGFβ1.

Fibroblasts play an essential role in maintaining the homeostasis of interstitial matrix and adjacent tissue under physiological condition in the kidneys. Although many types of kidney cell participate into the initiation and progression of kidney fibrosis, fibroblast is the major one for producing extracellular matrix through acquiring the phenotype of myofibroblast^1^,^26^,^27^,^28^. In this study, we found that endogenous n-3 PUFAs protects against kidney fibrosis in mice with UUO nephropathy. Furthermore, in cultured NRK-49F cells, DHA can remarkably suppress fibroblast activation triggered by TGFβ1. Regarding the critical role for fibroblast activation in kidney fibrosis, it may be concluded that n-3 PUFAs inhibits kidney fibrosis involving the suppression of fibroblast activation.

Tissue fibrosis and Inflammation are two closely inter-wined events during the progression of various types of CKD. Inhibition of inflammation may attenuate kidney fibrosis^28^,^29^,^30^. In this study, ureter obligation induced remarkable inflammatory cell accumulation in the wild type mice, whereas inflammatory cell accumulation was largely reduced in fat-1 trangenics. Anti-inflammatory property of n-3 PUFAs has been well documented by the previous studies^3^,^31^,^32^. It is known that membranous phospholipids serve as the primary reservoir for lipid inflammatory mediators. N-3 PUFAs may compete with n-6 PUFAs to suppress the synthesis of potent pro-inflammatory cytokines and produce anti-inflammatory catabolites in kidney diseases. Additionally, enrichment of n-3 PUFAs in the membrane of inflammatory cells may prohibit the cells to move to injured tissue. Therefore, n-3 PUFAs may inhibit inflammatory cell accumulation in the obstructive kidneys through decreasing the production of pro-inflammatory cytokines and retarding inflammatory cell migration, adhesion and migration. In this regard, n-3 PUFAs protect against kidney fibrosis through inhibiting both fibroblast activation and inflammatory cell accumulation.

Mammalian target of rapamycin (mTOR) is a serine/threonine protein kinase which regulates numerous biological processes, including cell metabolism, growth, proliferation, survival and aging^33^,^34^. MTOR exists in two structurally and functionally distinct complexes, named mTORC1 and mTORC2 respectively. Accumulated evidences suggest that mTOR may act as a central modulator in a variety of kidney disorders^35^,^36^,^37^,^38^. Except for being a reservoir for bioactive molecules within cellular membrane, n-3 PUFAs may also affect the function of membrane-embedded proteins, and thereby regulating a number of intracellular signaling pathways such as mTORC1 and mTORC2^39^,^40^,^41^. In this study, we found that DHA could reduce Akt phosphorylation at Ser473 but not S6 or Smad3 phosphorylation stimulated by TGFβ1 in NRK-49F cells, suggesting that n-3 PUFAs block mTORC2 but not mTORC1 or Smad signaling in this cell type. However, DHA may inhibit both mTORC1 and mTORC2 signaling in many types of tumor cell, which indicates that DHA may affect mTOR signaling at a cell context dependent manner^39^,^40^. Regarding the critical role for mTORC2 signaling in promoting fibroblast activation and kidney fibrosis, it may be concluded that n-3 PUFAs inhibits kidney fibroblast activation via mTORC2 signaling inhibition but independent of mTORC1 and Smad signaling. More work is needed to further elucidate the underlying mechanisms for n-3 PUFAs in inhibiting kidney fibroblast activation and kidney fibrosis. In this study, DHA failed to inhibit TGFβ1-induced Smad3 phosphorylation, a key mediator of TGFβ1 signaling, in NRK-49F cells^42^. Intriguingly, induction of endogenous n-3 PUFAs by fat-1 transgene attenuates Smad3 phorphoylation in the fibrotic kidneys after UUO, which may be the consequence of the less kidney injury after UUO in fat-1 transgenics compared to those in the wild types.

In summary, the present study verifies the role for n-3 PUFAs in protecting against kidney fibrosis and indicates that mTORC2 signaling may be the potential target for n-3 PUFAs in protecting against fibroblast activation and kidney fibrosis.

## Methods

### Mice and animal models

Fat-1 transgenic mice were generated in the C57BL/6 background, and the mice were derived from one of the previously characterized transgenic founders^43^. All animals were maintained in the specific pathogen-free Laboratory Animal Center of Nanjing Medical University according to the guidelines of the Institutional Animal Care and Use Committee at Nanjing Medical University, Nanjing, China. Genotyping was performed by PCR assay using DNA extracted from the mouse tail. The primers used for genotyping were as follows: fat-1 genotyping, sense: 5′-GGACCTGGTGAAGAGCATCCG-3′ and anti-sense: 5′-GCCGTCGCAGAAGCCAAAC-3′.

UUO was performed as previously reported^35^. The UUO kidneys were harvested at day 7 after surgery. One portion of the kidney was fixed in 10% phosphate-buffered formalin, followed by paraffin embedding for histological and immunohistochemical staining. Another portion was immediately frozen in Tissue-Tek optimum cutting temperature compound (Sakura Finetek, Torrance, CA) for cryosection. The remaining kidney tissue was snap-frozen in liquid nitrogen and stored at −80 °C for subsequent protein extraction.

All experiments were performed in accordance with the approved guidelines and regulations by the Animal Experimentation Ethics Committee at Nanjing Medical University. All experimental protocols were approved by the Animal Experimentation Ethics Committee at Nanjing Medical University.

### Cell culture and treatment

Rat kidney interstitial fibroblasts (NRK-49F cells) were obtained from American Type Culture Collection (Manassas, VA). Cells were cultured in Dulbecco’s modified Eagle’s medium/F12 medium supplemented with 10% fetal bovine serum (Invitrogen, Grand Island, NY). The cells were seeded on six-well culture plates to 60–70% confluence in complete medium containing 10% fetal bovine serum for 16 h and then changed to serum-free medium after washing twice with serum-free medium. NRK-49F cells were pre-incubated with DHA (cat: 1001970314, Sigma-Aldrich, St Louis, MO) for 30 mins, followed by treatment of recombinant human TGFβ1 (cat: 100-B-010-CF, R&D Systems, Minneapolis, MN) for different time points or dosage as indicated.

### Lipid extraction and fatty acid analysis

Fatty acids were extracted using a method reported previously with some modifications^44^. n-6/n-3 ratio was measured by gas chromatographic analysis. Briefly, about 0.8 cm mouse tails were homogenized in a mixture of methanol, chloroform and water. They were allowed to stand for 15 minutes. More chloroform and water were added and the samples were vortexed and centrifuged. The lower phase was removed and dried under nitrogen, before being resuspended in boron trifluoride methanol. The samples were heated at 90 °C for 30 minutes and extracted with pentane and water. The samples were centrifuged and the upper phase was dried under nitrogen. The samples were resuspended in heptane and injected into a capillary column (SP2380 105 m  × 0.53 mm × 0.20 μm, Supelco, Bellefonte, PA, USA). An Agilent 7890A (Agilent Technologies, Santa Clara, CA, USA) performed the gas chromatography. Total n-6 fatty acids was calculated from linoleic acid (LA, 18:2 n-6) and arachidonic acid (AA, 20:4 n-6). Total n-3 fatty acids were calculated from a-linolenic acid (ALA, 18:3 n-3), eicosapentaenoic acid (EPA, 20:5 n-3), docosapentaenoic acid (DPA, 22:5 n-3), and docosahexaenoic acid (DHA, 22:6 n-3).

### Histology and immunohistochemistry

Kidney samples were fixed in 10% neutral formalin and embedded in paraffin. 3 μm-thick sections were used for Periodic Acid–Schiff (PAS) and Masson staining. For immunohistochemical staining, paraffin embedded kidney sections were deparaffinized, hydrated, and antigen-retrieved. Endogenous peroxidase activity was quenched by 3% H2O2. Sections were then blocked with 10% normal donkey serum, followed by incubation with anti-type I collagen (cat: AB765P, Millipore, Billerica, MA) over night at 4 °C. After incubation of secondary antibody for 1 h, sections were incubated with ABC reagents for 1 h at room temperature before being subjected to substrate 3-amino-9-ethylcarbazole or 3,3′diaminobenzidine (Vector Laboratories, Burlingame, CA). Slides were viewed with a Nikon Eclipse 80imicroscope equipped with a digital camera (DS-Ri1, Nikon, Shanghai, China).

### Immunofluorescent staining

Kidney cryosections at 3 μm thickness were fixed with 4% paraformaldehyde for 15 min, followed by permeabilization with 0.2% Triton X-100 in phosphate-buffered saline (PBS) for 5 min at room temperature. After blocking with 2% donkey serum for 60 min, the slides were immune-stained with antibodies against FN (cat: F3648, Sigma-Aldrich, St Louis, MO), α-SMA (cat: A5228, Sigma-Aldrich, St Louis, MO) or F4/80 (cat: 14–4801, eBioscience, San Diego, CA). Cells cultured on coverslips were washed twice with cold 1 × PBS and fixed with cold methanol/acetone (1:1) for 10 min at −20 °C. After three extensive washings with 1 × PBS, cells were treated with 0.1% TritonX-100 for 5 min and blocked with 2% normal donkey serum in 1 × PBS buffer for 40 min at room temperature and incubated with anti-FN or anti-α-SMA, followed by staining with fluorescein isothiocyanate or tetramethylrhodamine-conjugated secondary antibody. Cells were also stained with 4′, 6-diamidino-2-phenylindole to visualize the nuclei. Slides were viewed with a Nikon Eclipse 80i Epi-fluorescence microscope equipped with a digital camera.

### Quantitative determination of total collagen and fibrotic area in kidney tissue

The total collagen in kidney tissues was quantitated as previously reported^35^. Briefly, 3-μm-thick sections of paraffin-embedded tissue were stained with Sirius red F3BA and Food Green FCF (Sigma-Aldrich) overnight. After washing three times with 1 × PBS, the dye was eluted from tissue sections with 0.1 N sodium hydroxide methanol. Absorbance at 540 nm and 605 nm were determined for Sirius red F3BA and Food Green FCF-binding protein, respectively. This assay provides a simple, relative measurement of the ratio of collagen to total protein, which is expressed as micrograms per milligram of total protein. As to the quantification of fibrotic area, kidney sections with Masson staining were used, and five randomly selected fields for each animal under 400 × microscope were quantified. The percentage of Masson-stained positive area was calculated using the Image J software (NIH, Besthesda, MD). Five individual animals for each group were used for matrix quantification.

### Western blotting analysis

Cultured NRK-49F cells were lysed in 1 × sodium dodecyl sulfate sample buffer. The kidneys were lysed with radio immunoprecipitation assay (PIPA) solution containing 1% NP40, 0.1%sodium dodecyl sulfate, 100 mg/ml phenylmethanesulfonylfluoride, 1% protease inhibitor cocktail, and 1% phosphatase I and II inhibitor cocktail (Sigma, St Louis, MO) on ice. The supernatants were collected after centrifugation at 13,000 g at 4 °C for 30 min. Protein concentration was determined by bicinchoninic acid protein assay (BCA Kit; Pierce Thermo-Scientific, Rockford, IL) according to the manufacturer’s instructions. Equal amount of protein was loaded into 10 or 12% sodium dodecyl sulfate-polyacrylamide gel electrophoresis (SDS-PAGE) and transferred onto polyvinylidene difluoride membranes. The primary antibodies were as follows: anti-GAPDH (cat: FL-335, Santa Cruz Biotechnology, Dallas, TX), anti-p-Akt (Ser473) (cat: 3868, Cell Signaling Technology), anti-p-Akt (Thr308) (cat: 2965, Cell Signaling Technology), anti-p-Smad3(Ser423/425) (cat: ab52903, Abcam), anti-FN (cat: F3648, Sigma-Aldrich), anti-α-SMA (cat: A5228, Sigma-Aldrich), and anti-type I collagen (cat: AB765P, Millipore). Quantification was performed by measuring the intensity of the signals with the aid of National Institutes of Health Image J software package.

### Data and statistical analysis

All data examined are presented as mean ± s.e.m. Statistical analysis of the data was performed using the SigmaStat software (Jandel Scientific Software, San Rafael, CA). Comparison between groups was made using one-way analysis of variance, followed by the Student–Newman–Keuls test. *P* < 0.05 was considered statistically significant.

## Additional Information

**How to cite this article**: Zeng, Z. *et al*. Omega-3 Polyunsaturated Fatty Acids Attenuate Fibroblast Activation and Kidney Fibrosis Involving MTORC2 Signaling Suppression. *Sci. Rep.* **7**, 46146; doi: 10.1038/srep46146 (2017).

**Publisher's note:** Springer Nature remains neutral with regard to jurisdictional claims in published maps and institutional affiliations.
