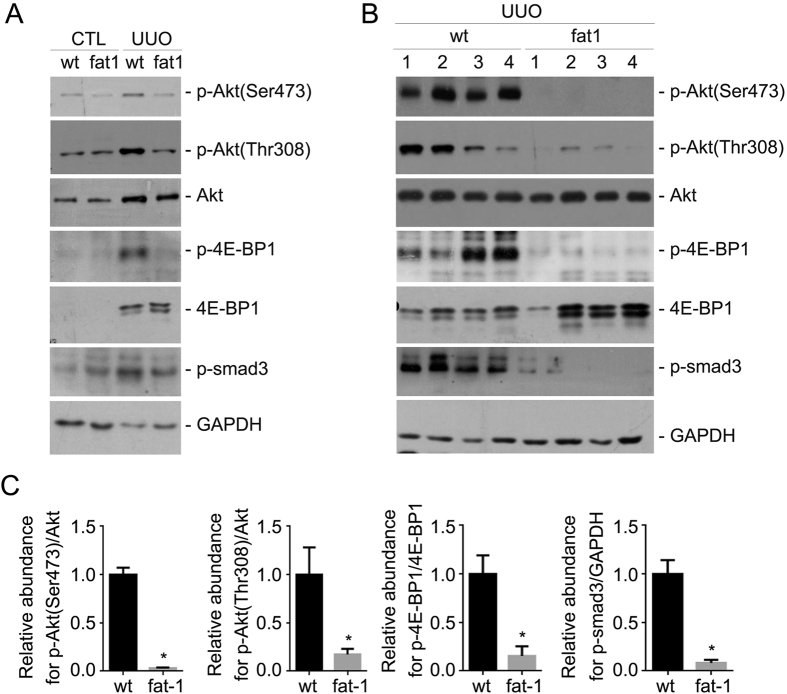# Corrigendum: Omega-3 Polyunsaturated Fatty Acids Attenuate Fibroblast Activation and Kidney Fibrosis Involving MTORC2 Signaling Suppression

**DOI:** 10.1038/srep46861

**Published:** 2017-06-26

**Authors:** Zhifeng Zeng, Haiyuan Yang, Ying Wang, Jiafa Ren, Yifan Dai, Chunsun Dai

Scientific Reports 7: Article number: 46146; 10.1038/srep46146 published online: 04 10 2017; updated: 06 26 2017.

This Article contains an error in Figure 6B, where the first sample of wt mouse for p-Akt (Thr308) should have been omitted. The correct Figure 6 appears below as Figure 1.

## Figures and Tables

**Figure 1:**